# “If You Don’t Do Parking Management .. Forget Your Behaviour Change, It’s Not Going to Work.”: Health and Transport Practitioner Perspectives on Workplace Active Travel Promotion

**DOI:** 10.1371/journal.pone.0170064

**Published:** 2017-01-30

**Authors:** Nick Petrunoff, Chris Rissel, Li Ming Wen

**Affiliations:** 1 Sydney School of Public Health, Sydney Medical School, University of Sydney, NSW, Australia; 2 Health Promotion Service, Sydney Local Health District, Camperdown, NSW, Australia; University of Edinburgh, UNITED KINGDOM

## Abstract

**Objectives:**

After having conducted two studies of the effectiveness of workplace travel plans for promoting active travel, we investigated health and transport practitioners’ perspectives on implementing workplace travel plans to share some of the lessons learnt. The objectives of this study were to describe perceived elements of effective workplace travel plans, barriers and enablers to workplace travel planning, their experiences of working with the other profession on travel plan implementation, their recommendations for workplace travel planning, and also to explore similarities and differences in transport and health practitioner perspectives.

**Materials and Methods:**

Fourteen health and ten transport practitioners who had prior involvement in workplace travel plan programs were purposefully selected from workplaces in Australia. We conducted 20 in-depth interviews since data saturation had been reached at this point, and data were subject to framework analysis.

**Results:**

Perceived essential elements of effective workplace travel plans included parking management; leadership, organisational commitment and governance; skills and other resources like a dedicated travel plan coordinator; and, pre-conditions including supportive transport infrastructure in the surrounds. Recommendations for promoting travel plans included supportive government policy, focusing on business benefits and working at different scales of implementation (e.g. single large worksites and business precincts). Health and transport practitioner perspectives differed, with transport practitioners believing that parking management is the key action for managing travel demand at a worksite.

**Conclusions:**

Health practitioners implementing travel plans may require training including concepts of travel demand management, and support from transport planners on parking management strategies. Promoting an understanding of the shared travel behaviour change skills of transport and health practitioners may assist further collaboration. For take-up by organisations to be of sufficient scale to create meaningful population level reductions in driving and increases in active travel, promotion and travel plans should be focused on the priorities of the organisations. Supportive government policy is also required.

## Introduction

Walking and cycling are active forms of transport. Public transport can also be considered active travel when the journey involves walking or cycling between the transport interchange and the destination. Workplace travel plans that promote active forms of transport as alternatives to driving private motor vehicles to work are site-based delivery mechanisms for transport demand management options.[1] They are also referred to as transportation demand management plans in North America and mobility management plans in Europe, and employ a mix of strategies including policy (e.g. parking policy, public transport discount schemes), infrastructure (e.g. end of trip facilities which include secure bicycle storage, showers and lockers) and behaviour change (e.g. referral to personal journey planning at staff induction, cycling and walking programs).

Insufficient physical activity is a risk factor for chronic health conditions and it is the fourth leading risk factor for death worldwide.[2] Epidemiological research suggests reducing car driving and increasing active travel for commuting results in overall increased physical activity,[3] is associated with decreased body weight and reduced risk of myocardial infarction in both cross-sectional, longitudinal and experimental studies.[3–16] This has included cross-sectional, longitudinal and experimental research specifically focused on commuting to work.[3, 9, 12, 13, 17–21]

Workplace travel plans could have important health benefits resulting from decreases in the proportion of workers using relatively sedentary forms of travel to work (driving private motor vehicles) and increases in workers choosing physically active modes of travel to work, which may also result in an overall increase in physical activity levels of the worker population, but research to demonstrate this effect is not conclusive. Published in 2013, a Cochrane review of organisational travel plans for improving health identified only 17 studies, five of which were conducted in workplaces. These controlled workplace studies investigated the effectiveness of single actions within travel plans, rather than an overall travel plan. The Cochrane review concluded that there was insufficient evidence at the time for the effectiveness of organisational travel plans for improving health.[22] A recently published systematic review of the effect of active travel interventions conducted in work settings on driving to work identified 12 controlled and longitudinal studies,[23] one of which was a workplace travel plan intervention which achieved a 42% decrease in driving alone at the intervention site and a 5% decrease at the control site with corresponding large increases in active travel to work. This study did not measure impacts on worker physical activity levels.[24]

The strongest available studies of the effect of workplace travel plans on worker health were not included in these reviews since they did not include controls sites. Both were time-series studies. The first used data from five bi-annual staff travel surveys of Bristol University staff over nine years. It found decreases in motorised transport corresponded with increases in walking in cycling to work each survey year over the nine-year period, and modelling from the final survey found usual walkers or cyclists were achieving over 80% of their weekly requirements for physical activity for health from their work commutes.[25] Subsequent to this study and the systematic reviews described above, results of the evaluation of a three-year workplace travel plan encouraging active travel to work demonstrated that 4–6% increases in worker active travel to work were achieved, and these results remained significant over two years. This three-year time-series study called for more research with stronger study designs on the effect of these promising interventions for increasing worker active travel and overall physical activity levels.[26]

In some jurisdictions, travel plans can be required through the land use planning and approvals process for new and expanded buildings.[27–30] However, the ad-hoc system of support for implementation of travel plans that are required by these mechanisms has been criticized for reasons including a lack of follow-up to ensure effective implementation, and responsibility for developing the travel plan often lying with developers rather than tenants who will occupy the building, which can lead to a ‘tick box’ approach to their development and poor implementation.[29, 31] The Australian national *TravelSmart* program and federal government funded *Healthy Worker Initiatives* in some states have both prompted population health professionals to support implementation of travel plans, although a review of the status of the national *TravelSmart* program in 2012 showed the level of support was mixed across Australian States and Territories.[32] Hospitals have also implemented travel plans in response to global *Green Hospitals* and *Healthy Hospitals* movements, or local sustainability initiatives.

We developed a workplace travel plan for a large outer-suburban hospital in Sydney, Australia, [33] validated survey measures to assess it’s main outcomes [34] and assessed the effectiveness of the four-year program.[26] We also conducted a secondary analysis of the results of a six year workplace travel plan program at two other large hospitals in Perth, Australia.[24] In keeping with good practice for evaluating complex health promotion programs, such as those recommended in Medical Research Council guidelines,[35] we included qualitative research reported in this paper to share the lessons learnt from these experiences as well as the lessons of other practitioners who had implemented comprehensive workplace travel plan programs.

Qualitative research has shown developers and owners of sites that are required to develop travel plans generally have a positive orientation to the concept and can develop them well.[36, 37] There are published ‘best practice’ cases where workplace travel plans have achieved large impacts on staff active travel and reduced driving to work.[24, 25, 38] What makes these travel plans effective has been described in reviews from the perspectives of ‘experts’ [38, 39] and quality check lists for implementation exist.[40] However, experts agree that travel plans are not being taken up by sufficient numbers of organisations, and improved support for organisations implementing travel plans is needed to achieve positive population level impacts on physical activity and traffic congestion, even in countries which have trialed supporting their implementation at scale.[41] Only one qualitative paper has focused on aspects of implementation, and this was for residential travel plans.[36]

Given there have been recent Australian policy initiatives where health practitioners have supported implementation of workplace travel plans, and since the perspectives of health and transport practitioners who have implemented travel plans aiming to improve health have never been documented, the purpose of our study is to describe these perspectives to inform policy and programs which support successful implementation of travel plans aiming to increase active travel to work. The specific research questions of this qualitative study are described in Table 1.

**Table 1:** Research questions addressed in the present study.

Research question
1. What do health and transport practitioners who have supported workplace travel plans describe as the essential elements of what makes implementation successful, unsuccessful and why?
2. What are the orientations of health and transport practitioners who have supported travel plans toward them as a mechanism for increasing active travel to work?
3. How do health and transport practitioners describe experiences of working together to support travel plan implementation?
4. What do health and transport practitioners who have supported implementation of travel plans believe is the future for travel planning?
5. Do these perspectives of health and transport practitioners who have been involved in supporting implementation of workplace travel plans vary?

## Materials and Methods

The Consolidated Criteria for Reporting Qualitative Research (COREQ) were used to guide the research methods and analysis of results.[42]

### Methodological approach

The methodology for this research is guided by phenomenological interpretive approaches, to examine the essential elements of the experience of implementing travel plans from the perspectives of health and transport practitioners who have been involved in supporting their implementation. Objectivity in this study meant ‘bracketing off’ the research team’s views and recognising that the subjective views of participants are their perceived reality, regardless of whether they occur objectively. This enhances the relevance of findings in this study to real world policy and practice provided important context is described.[43]

### Context of interviewees and interviewer

Participants’ experience with travel plans, whether they were health or transport practitioners and whether they were primarily involved with implementation of travel plans or making decisions around supporting their implementation are summarised in Table 2. Participants were selected from New South Wales (NSW) (15), Western Australia (7) and Victoria (2) to provide a range of Australian experiences.

**Table 2:** Interview participants’ professional background and their experience with supporting implementation of travel plans.

Interview, [Code]	Professional background	Health	Transport	Implementer	Decision-maker	Experience ^a^	Scale ^b^
1, [HI1]	Health Promotion.	X		X		M	A
2, [TI2]	Sustainability, Consultancy.		X	X		H	A-D
3, [HD3]	Hospital Executive.	X			X	None prior, M	A
4, [HD4]	Health Promotion.	X			X	None prior, M	A
5, [HI5]	Health Promotion.	X		X		None prior, L	A
6, [HI6]	Health Promotion.	X		X		None prior, L	A
7, [HD7]	Health Promotion.	X			X	None prior, L	D
8, [HI8]	Health Promotion.	X		X		None prior, L	D
9, [TD9]	Planning, Urban Planning, Transport Planning.		X		X	H	A-D
10, [TD10]	Engineering, Transport Planning, Academia.		X		X	H	A-D
11, [HI11]	Health Promotion, Planning.	X		X		M	A,C
12, [TI12]	Sustainability.		X	X		None prior, M	A
13, [TD13]	Transport Planning.		X		X	H	A-D
14, [HI14]	Health Promotion.	X		X		H	A,C
15, [HD15]	Health Promotion, Planning, Management.	X			X	H	A,C
16, [TI16]	Parking Management.		X	X		None prior, M	A
17, [HI17]	Health Promotion.	X		X		None prior, L	A
18, [HD18]	Population Health, Health Promotion, Planning.	X			X	H	A-D
19, [TI19]	Transport Planning, Consultancy.		X	X		H	A-D
20, [TD20]	Urban Planning, Transport Planning, Consultancy.		X		X	H	A-D
**TOTAL**		**12**	**8**	**11**	**9**		

^**a**^ Experience supporting travel plans–None = no experience supporting travel plans prior to the one they supported at or close to the time of being interviewed; L = low, <2 years; M = moderate, 2–5 years; H = high, > 5 years.

^**b**^ Scale of travel plan implementation supported–A = single site; B = precinct level Transport Management Association; C = multi-site or health service level; D = systematic and State/large jurisdiction level support.

The interviewer (NP) had over five years’ experience implementing workplace travel plans. He was not working with any of the participants at the time of the interviews.

### Participant selection method for qualitative interviews

Twenty-four participants were purposively selected to provide the perspectives of two sub-groups–transport practitioners and health practitioners—involved in the implementation of workplace travel plans aiming to improve the health of workers. This sample size was considered adequate because the nature of the topic was clear, the data provided by this purposive sample were likely to be high quality since over three-quarters of participants had medium or high levels of experience implementing travel plans (see Table 2) and it is generally accepted that 20–30 in-depth interviews provides enough information to explore a topic in depth.[44] None of the 24 invited interviewees declined to participate and four were not interviewed since the research team were satisfied saturation of themes had been reached prior to their scheduled interviews. These four individuals who were invited but were not interviewed were similarly balanced between health and transport practitioners, and all had either moderate or high levels of experience implementing travel plans. They were not interviewed since they had agreed to later dates for interviews than the participants, and saturation of themes was achieved prior to their scheduled interviews. Table 2 shows participant details.

The study was approved by the South Western Sydney Local Health District Human Research Ethics Committee. Participants provided written consent to participate.

### Methods for data collection and analysis

#### In-depth interview questions

A set of interview probes were formulated based on the research questions and organised into four topic areas, with prompts in each area (Table 3). A pilot interview was conducted to check that the probes were understood and elicited the types of information that addressed the research questions.

**Table 3:** Summary of interview prompts and probes for health and transport implementers and decision-makers.

The interview consisted of six sections.
**Background**
What led to your involvement in travel plans?
What is your experience relating to travel plans?
What is your current role with respect to travel plans?
**What works, what does not and why?**
Are you able to share some of the results you achieved?
Typically, in your view what things did the organisation(s) you worked with do well? What did they not do well?
Probe: Were there specific actions that were not completed? Why do you think this was the case?
**Orientation towards workplace travel plans**
What do you think of travel plans as a mechanism for increasing active travel to work?
**Health and transport practitioners working together**
Did a transport/health professional support the implementation of the workplace travel plan(s) you were involved with?
If yes, probe: What was the model for working with them? How were they involved in implementation?
Further probe: What was it like to work with a transport/health practitioner on the implementation of a workplace travel plan?
Further probes: Did the model for working together work well? Is there anything you would do differently?
**The future for travel planning**
What do you think the future directions could be for the travel plan(s) you have been involved in implementing?
How about workplace travel planning generally?
How about at a state level? Are workplace travel plans a strategy that justifies further investment by health/transport?
**Open prompt**
Is there anything else you would like to add?

#### Interview data collection

In depth interviews were conducted by one interviewer (NP). The interviewer had prior experience in conducting interviews, focus groups and observation methods guided via one-year of formal mentoring relationships with experienced qualitative researchers in an academic institution initially and as part of his employment in health and non-health settings.

Where possible, interviews were conducted face to face in a private meeting room at the interviewees place of work. In some instances, they were conducted over telephone or Skype and recorded. The interviewer transcribed the recordings after the interviews, using transcription software (F4transkript v5.60.3) to ensure word for word accuracy. Some participants checked the data syntheses to ensure they maintained anonymity.

#### Interview data coding and analysis

Framework analysis was chosen for its relevance to research intended to inform policy.[45] The steps for framework and thematic analysis are described briefly here and in detail elsewhere.[43, 45].

1. Familiarisation—two investigators (NP, CR) read the transcripts and field notes (NP repeatedly), recording initial impressions they presented to each other.
2. Thematic framework—themes were identified and a coding scheme was developed by the same two investigators.
3. Indexing—codes were applied to the whole data set in a systematic way by the two investigators who checked for discrepancies in coding before agreeing which code would be applied. The third investigator (LMW) checked these codes to ensure they reflected the data accurately.
4. Charting–the lead investigator rearranged summaries of the data by code and divided into health and transport sub-groups with a check box indicating implementer or decision maker status in tabular formats in a spreadsheet package with page references to the original transcripts; this enabled investigators to view the data across participants and down themes as shown in the supplementary file S1 Tables
5. Mapping and Interpretation–the charts were used to explore the meaning of the data, and look for similarities and differences in the discussion from health and transport sub-groups as well as implementer- and decision maker sub-groups.

The mapping and interpretation step involved further classification of the data, collapsing some into combined themes, creating explanatory accounts by looking for links or connections in the data and attachments to sub-groups. Finally, higher level explanations were developed after assessing the implicit and explicit meanings within the data and drawing on other empirical research findings, assessing links to theoretical frameworks and by considering wider explanations.

To increase accuracy and credibility of data analysis themes which emerged were checked for deviant cases. Enough context was provided for readers to judge the information presented and the original charting of the synthesis of the data is provided in a supplementary file which readers can examine. S1 Tables

## Results

The participants were categorised as health or transport decision-makers and implementers. Their professional backgrounds and experience supporting implementation of workplace travel plans are detailed in Table 2. The thematic findings are summarised below in relation to the research questions.

### What do health and transport practitioners who have supported workplace travel plans describe as the essential elements of what makes implementation successful, not successful and why?

The three prompts for participants to provide information on what they felt the essential elements of what makes travel planning successful generated discussion which was categorized into two main topic areas– 1) Parking, and 2) Barriers and enablers to travel planning. The topic of parking generated the largest amount of discussion, and this discussion differed between health and transport practitioners since transport practitioners often referred to it being the most essential action within travel plans. The three themes which arose included travel demand management and parking; parking management as a challenging action to implement in travel plans; and, enablers to implementing parking policy. The broad topic of barriers and enablers to travel planning included three themes of leadership, organizational commitment and governance; skills and other resources; and, pre-conditions for successful travel planning.

### Topic 1 –Parking

#### Travel demand management and parking

Parking was often referred to explicitly as a central component of site level travel demand management in workplace travel planning, mostly by transport practitioners. Talking about key learnings from supporting implementation of travel plans, a transport decision-maker states: *"I think the key thing is not to consider things in isolation*. *If you don’t do parking management*, *you know*, *forget your behavior change*, *it’s not going to work"*.[TD9] In contrast two health decision makers felt that their experience demonstrated you can still get small but meaningful change without this “key” action. [HD4, HD15]

#### Parking management is a challenging action to implement in travel plans

Responses highlighted practical issues like requiring long lead times, being more difficult in sites where free parking has existed or it is in employment contracts, health facilities having a high proportion of staff who are shift workers and challenges for health leadership addressing union perceptions that parking is *“an essential service”* for employees. [TD9, HD3] One health decision-maker felt this perception was the main barrier to successful travel plan implementation:

*“*..*I think the main barrier is*.. *the overriding mindset that we have to accommodate cars for everybody*.. *a travel plan*.. *starts to challenge that paradigm*.. *and that’s the thing that developers or hospitals and organisations don’t do well because they meet the perception*.. *if you build the infrastructure pretty much for anything if it’s planned well enough*, *people will use it*.. *if you build big car parks then people will drive*..*”* [HD15]

Participants often spoke about parking being considered a right instead of a privilege by employees and unions, and something human resource managers felt necessary to attract employees.

#### Enablers to implementing parking policy

Health and transport practitioners both discussed enablers to implementing parking policy, with transport practitioners expressing control of the issue. When discussing the Western Australian experience of creating and implementing mandatory parking policy for all health campuses which caps the number of parking spaces at a site and specifies criteria which prioritises parking for employees who need it, one decision-maker stated: *“*..*All the arguments that it’s all too hard*, *etc*. *are rubbish*.*”* Later stating, *"*..*you have to present the other elements of the behaviour change*.. *strategies*. *You have to present a complete package*.*"* [TD9] This implies that strategies to encourage alternative forms of travel to car driving offset strategies to discourage driving such as parking restrictions, parking prioritisation based on need and price increases, making the overall package of strategies acceptable to employees. Enablers to parking policy participants mentioned are presented in Table 4.

**Table 4:** Enablers for parking policy actions in workplace travel plans.

Code ^a^	Enabler
HI1, TI12, TI16	Loss of parking spaces at inner city hospital sites to accommodate more services forcing hospital management to address parking issues.
HD3	NSW Ministry of Health parking policy currently being an impediment to promoting active travel, but also an opportunity to advocate for changes that will create a supportive policy framework in the future.
HD15	Framing travel plans as a solution to parking problems rather than stating parking is being reduced.
TD9	Presenting strategies to discourage driving as a package with strategies that encourage active travel.
TD9, TD13	Pricing parking at it's true cost, capping spaces and running it as a business being the solution to making all transport at a site efficient.
TD9	Running parking as a business where the true costs of supplying parking are accounted for it to be priced appropriately to make a profit, and requiring this in approvals for expansion.
TD2, TD3, TD4	Parking management policies allocating revenue from parking to incentives for active travel such as increases to public transport levels of service (e.g. a new bus service) or new infrastructure supporting walking and cycling.
TI2	Parking policy including a prioritisation scheme, with eligible employees being able to cash out their parking and use it to fund alternative transport.
TD9, TD13	Fringe benefits tax being triggered at sites in some instances where employees are being provided parking for free or at discounted rates.
TD9, TD10	In central locations where parking levies exist, parking must be priced and funds used for travel demand management strategies at a regional level.
TD20	Parking “cash out” programs (refers to an off the shelf package) incentivise employees not driving whilst helping employees to understand the true cost of providing parking.
TD13	Easier at new sites, difficult when free parking existed previously.

^**a**^ Interview participant code from column one of Table 2.

### Topic 2—Barriers and enablers to travel planning

#### Leadership, organisational commitment and governance

A common point for discussion was that whilst leadership in an organisation implementing a travel plan needs to be both top down and bottom up, top down leadership is critical to getting contested actions implemented. There was passionate discussion of leadership (or its absence) at state government level on policy actions to support travel plans and other strategies to promote active travel as an alternative to driving cars. Discussion about organisational commitment often referenced specific actions which either demonstrate commitment or a lack thereof (e.g. building actions into operations like new staff induction) and a lack of commitment was cited as a common source of implementation failure. Good governance for the travel plan was seen as critical and documenting the actions in the travel plan itself was spoken of as an important mechanism for reporting progress and re-gaining commitment.

#### Skills and other resources

A mix of discussion occurred under this theme. The types of skills required to support workplace travel plan implementation were spoken about. Two health practitioners felt that whilst the skill sets of health promotion professionals matched those required to supporting common actions in workplace travel plans, some would need training in the content areas of active travel and travel planning. [HI1, HI8] One transport practitioner who was a decision-maker felt that travel demand management and travel behaviour change were concepts that people in local government responsible for supporting travel plans needed to be trained in. [TD9] Common discussion about resources included that it would be ideal to have parking revenue fund actions in travel plans and the importance of adequate funds for strong encouragement strategies (e.g. public transport ticket subsidies, bike loan schemes). One transport decision maker stated that this was a key learning, *"*..*yes if it's not someone's job*, *in time it risks not getting done*.*"* [TD13] This quote is reflective of the most common suggestion under this theme that a permanent and dedicated coordination position was important.

#### Pre-conditions for successful travel plan implementation

Whilst there was some discussion about operations being suitable (e.g. organisations being able to allocate parking revenue to travel plan actions), most discussion related to transport infrastructure: *“*..*and the real ticket for the higher sustainable transport use is to have the infrastructure supporting operations in place*..*”* [TD20]

At a site level this was about end of trip facilities and in the surrounding area this focused on public transport access and levels of service as well as a connected and complete walking and cycling network.

### What are the orientations of health and transport practitioners who have supported travel plans toward them as a mechanism for increasing active travel to work?

When participants were prompted to discuss what they thought of travel plans as a mechanism for increasing active travel to work most participants acknowledged they can be effective for increasing worker active travel. A clear, single theme arose from two discussion points which often followed one another—physical activity by stealth and the challenge of selling active travel plans. This is presented below under the third topic area of workplace travel planning and active travel.

### Topic 3—Workplace travel planning and active travel

#### Physical activity by stealth–the challenge of selling active travel plans

Participants understood that increasing active travel to work is not the main reason a business would choose to develop a travel plan, but increased employee physical activity is a serendipitous benefit of developing one. One transport decision-maker stated:

*"*.. *health is just not even on the radar for people doing a travel plan*. *It’s about*.. *parking pressure*, *or staff retention*.. *So unless you are able to draw those dots together through something that starts as cost*, *but turns out as a health benefit*..*"* [TD20]

In the context of state/national programs which include support for businesses developing travel plans delivered via government agencies including health, participants felt that recognising the motivations of organisations considering implementing a travel plan helped gain their commitment.

There was also a feeling that there was some work to do, within health and other government agencies at all levels, selling active travel and travel planning since “..it’s no one’s core business.” [HD18]

### How do health and transport practitioners describe experiences of working together to support travel plan implementation?

Participants from health and transport disciplines provided descriptions of working together with the other discipline to implement travel plans in different ways. Under the fourth topic area of health and transport working together two themes which reflect participants’ experiences were a different level of understanding of each other’s skills and collaboration.

### Topic 4—Health and transport working together

#### A different level of understanding of each other’s skill sets

Health practitioners clearly described the skills transport practitioners apply when supporting implementation of travel plans and acknowledged their expertise was needed for particular actions (e.g. addressing parking). Two experienced health promotion practitioners who had worked with transport practitioners to support travel plan implementation stated explicitly that transport practitioners did not understand their skill sets:

*“Um*, *I don’t necessarily think that the Transport people understand how well that Health does behavior change*. *And so*.. *they don’t see health as*, *as strong*.. *a collaborator as health is*…*They really do share some common skills but they just don’t know”*. [HD18]

Transport practitioners recalled experiences of implementing travel plans for health facilities where gaining end-user clinical health staff input was valuable since it ensured their needs were met. Two also recalled experiences working with health bureaucrats to develop parking policy for health facilities, which was made difficult by union involvement.

#### Collaboration

Most discussion relating to collaboration was from health practitioners. Experiences were mostly positive and participants felt more collaboration would be beneficial. Two health practitioners suggested collaboration with local government is an essential action in travel plans. One health decision-maker stated:

*“*..*There’s*.. *bits and pieces of engagement around between transport and health*. *I think that could be really formalised with some dedicated action*,..*that’s feeding into state plan delivery targets and can be jointly funded for benefit across both agencies*. *It*.. *will require time and that’s one of the challenges*.*”* [HD18]

Another health decision-maker believed the concept of integrated land use and transport planning was bringing the disciplines of transport and planning closer together, making it easier for health to collaborate since historically urban planners had ties with public health. [HD15]

### What do health and transport practitioners who have supported implementation of travel plans believe is the future for travel planning?

Participants spoke passionately about what they felt the future may be for workplace travel planning, providing many recommendations. This fifth topic area of the future of workplace travel planning generated two themes of policy plus working at different scales and implementation support.

### Topic 5—The future for workplace travel planning

#### Policy

When discussing the future for workplace travel planning both health and transport practitioners spoke about policy. Overwhelmingly, participants felt that policy requiring development of travel plans was a necessary “trigger” for their development, and that improved strategies for supporting their implementation were needed. Specific examples of these suggested improvements are included in the full report of this study S1 Report.

#### Work at different scales and implementation support

When discussing the future of workplace travel planning there was a large amount of discussion from all categories of participants about working at different scales (e.g. regional, state, etc.), and this discussion was often intertwined with talk about how implementation at different scales could be improved, where this work was already occurring, or supported well. The broad range of scales and the types of implementation support suggested are included in a full report of this study S1 Report. A common suggestion was to focus recruitment and implementation support strategies for businesses implementing travel plans in regions where there were plans for significant upgrades to transport infrastructure. One participant concluded their feedback on the future for travel planning by stating: *"It’s a golden opportunity*.. *extend the reach and*.. *benefits of the Premier’s vision of all of this infrastructure change*.. *with behaviour change programs*.*"* [HD18]

### Do the perspectives of health and transport practitioners who have been involved in supporting implementation of workplace travel plans vary?

There were many differences in health and transport practitioner perspectives. These are described under topic six below, and the implications are considered in the discussion. These differences are important to consider for potential future collaboration between the disciplines to be successful at increasing the population levels of active travel.

### Topic 6—Similarities and differences in the perspectives of health and transport practitioners

There were some notable differences in the perspectives of health and transport practitioners on implementing workplace travel plans. These differences include, 1) the importance of parking management actions in travel plans as a way of managing overall travel demand at a site being discussed in a lot of detail mostly by transport practitioners; 2) a couple of health decision-makers felt that their experience demonstrated small but meaningful change is achievable without parking management actions in travel plans; 3) transport practitioners expressing control of the issue of parking management; 4) a different level of understanding of each other’s skill sets, where health practitioners felt their skills relating to behaviour change were not well understood by transport practitioners; and, 5) most discussion relating to collaboration between health and transport being from health practitioners. These differences are considered further in discussion.

## Discussion

### Travel demand management and parking

Most transport practitioners described parking as an essential component of managing travel demand at a site, whilst two health practitioners described it as a key action. Many health practitioners promoting active travel and supporting travel plans would not routinely be exposed to concepts of management of travel supply and demand in their training, therefore it is important that health practitioners understand these concepts for successful collaboration with transport practitioners on initiatives which aim to increase active travel. A text book on mobility management, sustainable transport and travel plans describes these concepts succinctly with sufficient background for health practitioners to understand them.[1] In simplified terms, supply side strategies for managing transport have traditionally emphasised planning for a single mode (motor vehicles) and planning has been characterised by a ‘predict and provide’ approach to providing infrastructure (car parking, roads) where growth is predicted and infrastructure is built to meet that future need. The demand focused tradition of transport planning emphasises multi-modality and managing existing resources efficiently.

A recently published case representation of lessons from five major cities in Germany, Austria and Switzerland documented the importance of this multi-modal approach in achieving significant reductions in car dependence in these cities.[46] Although the overall mix of policies varied, all five of the cities implemented roughly the same policies to promote walking, foster compact mixed-use development, and discourage car use. Of the car-restrictive policies, the authors stated that parking management at a city level was by far the most important. This finding supports and complements the findings in the paper presented here, that worksite level parking management was judged the most important strategy for managing travel demand at worksites implementing travel plans.

It is also possible that transport practitioners emphasised the importance of parking policy for managing travel demand since the concept of ‘induced traffic’ is well understood in transport circles, and evidence suggests more traffic is generated as a result of supplying more parking and road infrastructure, since driving becomes convenient.[47] Recognising the adverse consequences of this induced traffic has been an impetus for change in approaches to policy influencing transport supply and demand, since it provides a strong rationale for the ‘predict and provide’ approach being unsustainable.[47]

### Enablers to parking policy and managing travel demand

Whilst a common theme raised was that parking management is difficult to implement, participants also spoke about many enablers for implementing parking management strategies. One of these enablers, the approach of presenting a package of transport choices, described by practitioners in this study is consistent with recent best practice travel planning. Most transport practitioners and some health decision-makers suggested transport management strategies should not be considered ‘in isolation’. Packaged approaches usually combine ‘carrots and sticks’: where ‘carrots’ encourage alternatives to driving private motor vehicles and ‘sticks’, which discourage driving, are introduced simultaneously to increase their acceptability. Published literature suggests the most successful travel plans have adopted this approach.[24, 25, 38] Another approach some transport planners have considered is ‘phasing’ in transport supply and demand so demand side strategies are timed to complement supply of new transport infrastructure (particularly multi-modal networks).[48]

Understanding these concepts and how they can be translated to large scale implementation is critical to non-transport practitioners and policy makers being effective in achieving policy goals such as population level increases in active travel, large reductions in transport-related carbon emissions, decreased local road congestion or decreased parking pressure at sites. The best available evidence for travel behaviour change suggests that not considering these concepts will achieve modest changes at best.[49]

A study which aimed to understand 20 successful travel plans in the UK supports the participants’ view in this study that parking may be the critical success factor. It found that organisations that addressed parking in some way had more than double the reduction in driving (and shifts towards active travel) than those which had not. It provided many suggestions for increasing the acceptability of parking management strategies, but most of these organisations were from the private sector, and strategies for achieving this success may not be transferable to other sectors (e.g. financial incentives for not driving).[38] A review found that removal or reduction of parking subsidies had a strong effect on reducing solo-driving to work,[50] and a controlled experiment in the USA has shown that parking cash-out schemes are effective at reducing employees the solo-driver share of trips and increasing transit journeys and walking/cycling to worksites.[51] There is a published example of a large public sector organisation achieving large reductions in staff driving to work following the implementation of a travel plan which included parking management strategies. These strategies were made acceptable to staff using low cost methods including an off-site ‘park and ride’ facility (i.e. a parking station outside the city center to park a car or bike before taking public transport, walking or cycling for the remainder of the journey) and collaborating with local government to increase levels of public transport services between the work site and this ‘park and ride’ facility. [24]

A qualitative study including 32 semi-structured interviews and review of policy documents to describe the city of Vienna’s achieving the largest reductions in motor vehicle use amongst European cities who measured this between 1990 and 2015. A wide range of politicians, transport planners, and academics almost unanimously identified the expansion of the U-Bahn (metro) and parking management as the most important policies accounting for the reduction in car mode share since 1993. This city-level achievement provides some parallel evidence for the worksite-level findings in the study presented in this paper that parking management is a critical factor in achieving shifts from car driving towards walking, cycling and public transport use. It also describes the long term political and social processes that were required to generate the political will for this contentious parking policy to become mainstream, which included local district pilots and subsequent experiments to demonstrate it was working to relieve traffic congestion and parking pressure, whilst providing a package of excellent alternatives to car driving to increase the acceptability of parking policy.[52]

Some health decision-makers felt that their experience with implementing travel plans had shown small but meaningful increases in active travel to work can be achieved without addressing parking. This belief may stem from principles of translating empirical research through to meaningful population level health gains, where small impacts across large populations are important.[53] However, to achieve large population reach organisational travel plans need to be adopted by many organisations, which requires consideration of what motivates most organisations to develop travel plans and what they would define as meaningful change.[54] A growing organisation in a constrained space (e.g. a large metropolitan hospital) may be motivated to commit to implementing a travel plan if it could avoid supplying another expensive multi-storey car park in future. Large reductions in staff driving to work would be required for the hospital to be able to avoid the need to build another large car park. The evidence to date suggests can only be achieved by travel plans which include parking management actions.[24, 25, 38]

Leadership, organisational commitment and governance were described as essential elements of what makes travel plans effective. This is consistent with the literature on successful travel plan implementation.[41] Skills and other resources were also described as enablers and a dedicated coordination position was a common response, which has also been described by others as a key success factor.[55]

### Travel plans as a mechanism for increasing active travel to work

Consistent with a growing body of literature on their effectiveness, there was consensus among health and transport practitioners that travel plans were effective for increasing active travel to work.[24–26, 38] However, discussion reflected the difficulty of getting more organisations to adopt them which has also been acknowledged in the literature on travel planning.[41] The inter-related themes participants discussed of travel plans achieving increases in employee physical activity levels by stealth, and how active travel is marketed both have implications for strategies to increase the take up of travel plans by organisations since they need to be sold to organisations as a solution for issues that are important to them. Whilst health may support travel plans to increase active travel and local government to decrease local traffic congestion, processes which encourage or require businesses to develop travel plans should focus on language that appeals to most organisations, and provide hard evidence or case studies of how they achieve these desired benefits for organisations. This may include improved site access for patients and customers, reduced transport or parking related costs, increased parking profits, increased business efficiency and improved staff satisfaction. When the link between travel plans and achieving health outcomes is made, it could be linked to arguments for improvements to work related outcomes including sickness levels/absenteeism and productivity when this evidence becomes available.

### Working together to support travel plan implementation

Health, or more specifically health promotion, practitioners’ comments reflected the belief that their skills are highly aligned to the skills required for travel behaviour change but they felt transport practitioners did not understand or appreciate this. Core competencies for health promotion, such as communication and partnership building, do in fact align well with the new skills required for planning, implementing and monitoring travel demand management strategies and supporting sustainable travel behaviour.[56, 57] Transport experts have described skills including marketing, communication and business management being important for travel demand management in contrast to engineering and operations skills required for supplying transport infrastructure.[48] Consequently, different people may be required in the processes of managing travel demand. Promoting an understanding of these shared skilled sets may assist with potential collaboration between health promotion professionals and transport planners.

### The future: recommendations for travel planning

Participants felt strong government policy support, including mandatory requirements for travel plans for some organsiations, was important for the future of travel planning. This discussion was strongly influenced by the policy context in participants’ home states. Some participants from NSW discussed the need for better support of the regulatory mechanism requiring travel plans to be developed as a condition of planning consent.[29] In Western Australia, where this regulatory mechanism did not exist at the time of these interviews, participants felt that whilst this regulatory requirement can result in a tick-box approach to developing travel plans it should still be adopted in that State with more thought given to how implementation and monitoring is supported. Whilst the literature shows that where the regulatory requirement exists it is the main reason businesses adopt travel plans,[1] it does not necessarily result in widespread adoption or effective implementation without suitable accountability measures.[29] Given the potential large benefits to transport systems and population health the issue of how implementation can be supported effectively deserves further applied intervention research and this could be informed by implementation theory and planning enforcement theory. It has been posited that a combination of these theoretical approaches to supporting policy requiring organisations to develop travel plans would result in successful support of implementation.[36] Consideration should also be given to pre-conditions for successful travel plan implementation described in this study and other literature.[38, 41]

In Western Australia, the Access and Parking Strategy for Health Campuses in the Perth Metropolitan Area mandates that with few exemptions, all health facilities adopt travel plans.[2] This policy states that: “Each health campus will develop a travel plan to meet the access needs of patients, visitors and employees. Revenue raised from parking charges may be used to fund the identified travel plan initiatives.” It specifies how the overall number of parking spaces at a site should be limited, and includes information to assist development of parking prioritisation systems which ensure staff and patients who need access to parking receive priority. Two participants referred to this policy as a model for other States to adopt. Indeed, in NSW some health decision-makers in this study referred to the current State level parking policy for health facilities as an opportunity for creating a supportive policy framework for reducing staff driving to work and increasing staff active travel. Such policy examples have relevance to many regions where driving motor vehicles is the dominant form of travel to work and organisations provide parking for workers. The Perth example has direct relevance to hospitals in many countries.

Work at different scales was another prominent theme referred to by participants talking about the future for travel plans. Complementing behaviour change with new infrastructure projects is currently receiving research attention. Several studies have focused on new cycleways or busways, and two of these have attempted to measure the effect of behaviour change programs on their use.[58–61] Some very new research has also focused on the effect of transport networks on active travel.[62] There is great potential to also measure the impacts of complementary travel demand management initiatives, including workplace travel plans, on the use of transport infrastructure and networks at a regional scale. Research funding agencies should consider funding this research.

### Similarities and differences in the perspectives of health and transport practitioners

Whilst the discussion from all participants relating to themes of barriers and enablers and travel plans as a mechanism for increasing active travel to work was similar, most of the perspectives shared by transport and health professionals were markedly different. Differences already discussed above include the importance of parking management actions in travel plans as a way of managing overall travel demand at a site being discussed in a lot of detail, and mostly by transport practitioners; two health decision-makers felt that their experience demonstrated you can still get small but meaningful change without parking management actions in travel plans; and, a different level of understanding of each other’s skill sets, where health practitioners felt their skills relating to behaviour change were not well understood by transport practitioners.

Two differences not discussed above include transport practitioners expressing control of the issue of parking management, and most discussion relating to collaboration between health and transport being from health practitioners. The sense of control of the issue of parking management expressed by transport practitioners may relate to many of these practitioners having the training and skills required to develop parking management plans, but responses were also influenced by context since some Western Australian interview participants worked on hospital travel plans and there was policy which made it mandatory for public hospitals in Perth to develop travel plans which addressed parking.

Most discussion relating to collaboration being generated by health practitioners may also have been influenced by context. Despite purposively sampling transport practitioners who had worked with health practitioners on implementing travel plans, transport practitioners did not refer to actions in travel plans being implemented by health practitioners (e.g. health promotion officers), and instead mostly referred to staff in health facilities they had implemented travel plans in being consulted whilst developing the plans, which is good practice. For some of the respondents this can be explained by them not having had the experience of truly collaborating with health practitioners on implementing travel plan actions. Where this type of experience was mentioned by two transport decision-makers they both stated the experience was positive and there should be more collaboration between health promotion practitioners and transport practitioners on the implementation of travel plans and other travel behaviour change initiatives. It may also be explained since developing partnerships is a core competency of health promotion practice, resulting in the health promotion practitioners interviewed being more likely to discuss this as part of their way of supporting travel plan implementation.[56] Addressing this lack of a shared understanding of each other’s skills could be done systematically by including information on this and the potential for collaboration in the university training of transport planners and in courses contributing to continuing education credits for relevant transport and planning professions.

### Limitations and strengths

Limitations of the research include that some responses under certain topics were influenced by the context at that point in time. The stage of development of workplace travel plans as an industry in NSW is relatively young. It has received relatively little support from state transport agencies, although some support is only now commencing in Sydney under the *Travel Choices* program.[63] Travel plan resources and cases have been promoted by the Premier’s Council for Active Living website,[64] and have recently received some support via the inclusion of travel planning in the *NSW Get Healthy at Work* program.[65] However, since health departments in many jurisdictions are not the lead agency for supporting implementation of workplace travel plans the perspectives of health practitioners presented in this study are likely to be relevant to the situation in Australia generally. As is recommended in qualitative studies, some context has been described in methods to assist the reader interpreting the findings.[42]

Strengths of the current study include using framework analysis to facilitate transparent and rigorous interrogation of the data. This analysis method was developed specifically for policy-relevant research and it has been used to produce original comparisons of health and transport practitioners’ perspectives to inform inter-disciplinary policy and practice.

## Conclusions

Health practitioners, such as health promotion professionals, supporting travel plan implementation require further training on key elements of effective workplace travel plans and concepts of travel supply and demand management. Promoting an understanding of the shared skills sets transport planners and health promotion practitioners have relating to travel behaviour change would assist further collaboration on support of travel plans and other travel behaviour change strategies, and would be beneficial for achieving large population increases in active travel. For take-up of travel plans by organisations to be of sufficient scale to achieve health and transport policy goals practitioners believe promotion and travel plans themselves should be re-oriented to focus on the priorities of the organisations rather than the policy objectives of government agencies (which will be achieved by stealth). Supportive government policy, with attention given to implementation support for travel pans, is also required and specific policy examples included the Western Australian Access and Parking Strategy for Health Campuses in the Perth Metropolitan Area being a model for other States and Territories to consider. A practical recommendation is to focus enhanced support efforts for organisations implementing travel plans in areas where upgrades to surrounding transport infrastructure are occurring. A research opportunity exists to test the combined effect of travel demand management strategies including travel plans with upgrades to transport infrastructure on traffic and active travel at a regional level and research funding agencies could consider funding such research.

## Supporting Information

S1 Report Full study report. (PDF)

S1 Tables Charts used for framework analysis. (XLSX)
